# Marine Protected Area Networks: Assessing Whether the Whole Is Greater than the Sum of Its Parts

**DOI:** 10.1371/journal.pone.0102298

**Published:** 2014-08-01

**Authors:** Kirsten Grorud-Colvert, Joachim Claudet, Brian N. Tissot, Jennifer E. Caselle, Mark H. Carr, Jon C. Day, Alan M. Friedlander, Sarah E. Lester, Thierry Lison de Loma, Daniel Malone, William J. Walsh

**Affiliations:** 1 Department of Integrative Biology, Oregon State University, Corvallis, Oregon, United States of America; 2 National Center for Scientific Research (CNRS), University of Perpignan, Perpignan cedex, France; 3 Laboratoire d'Excellence ‘CORAIL’, Perpignan cedex, France; 4 Humboldt State University Marine Laboratory, Trinidad, California, United States of America; 5 Marine Science Institute, University of California Santa Barbara, Santa Barbara, California, United States of America; 6 Department of Ecology and Evolutionary Biology, Long Marine Lab, University of California Santa Cruz, Santa Cruz, California, United States of America; 7 Great Barrier Reef Marine Park Authority, Townsville, Queensland, Australia; 8 Department of Biology, University of Hawai'i, Honolulu, Hawai'i, United States of America; 9 Marine Science Institute, University of California Santa Barbara, Santa Barbara, California, United States of America; 10 Centre de Recherches Insulaires et Observatoire de l'Environnement (CRIOBE), Moorea, French Polynesia; 11 Institute of Marine Sciences, Long Marine Lab, University of California Santa Cruz, Santa Cruz, California, United States of America; 12 Hawai'i Division of Aquatic Resources, Kailua-Kona, Hawai'i, United States of America; Universidad de Zarazoga, Spain

## Abstract

Anthropogenic impacts are increasingly affecting the world's oceans. Networks of marine protected areas (MPAs) provide an option for increasing the ecological and economic benefits often provided by single MPAs. It is vital to empirically assess the effects of MPA networks and to prioritize the monitoring data necessary to explain those effects. We summarize the types of MPA networks based on their intended management outcomes and illustrate a framework for evaluating whether a connectivity network is providing an outcome greater than the sum of individual MPA effects. We use an analysis of an MPA network in Hawai'i to compare networked MPAs to non-networked MPAs to demonstrate results consistent with a network effect. We assert that planning processes for MPA networks should identify their intended outcomes while also employing coupled field monitoring-simulation modeling approaches, a powerful way to prioritize the most relevant monitoring data for empirically assessing MPA network performance.

## Introduction

Anthropogenic impacts are increasingly modifying our oceans [1], subjecting marine ecosystems to threats ranging from climate change to pollution to overfishing [2], [3]. As a result, no-take marine reserves and other types of marine protected areas (MPAs) have been recommended as one tool to conserve marine biodiversity, ecosystem function, and the goods and services provided by healthy ecosystems [4], [5], [6]. Growing scientific information has shown that no-take marine reserves can provide benefits for adjoining fished areas [7], [8], [9] and serve as experimental controls for evaluating the impact of extractive activities on marine ecosystems and for distinguishing such effects from a changing global climate [10]. Full protection inside marine reserves has often led to consistent increases in species density, biomass, size, and diversity, with these results spanning diverse regions and reserves of varying sizes and ages (e.g., [11], [12], [13], [14] but see [15]). However, most of these data are from individual marine reserves, or groups of reserves [7], [8], which are each compared separately. To date, there is little evidence that MPAs in a given network are performing synergistically.

Properly designed networks of MPAs can theoretically outperform single marine reserves for a variety of ecological, economic, and social management goals. In theory, MPA networks can minimize the potential negative economic, social, and cultural impacts of a single large no-take reserve while producing similar or even greater ecological and economic returns from fishing outside the no-take areas (e.g., [16], [17], [18]). The International Union for Conservation of Nature's Marine Program defines a network as “a collection of individual marine protected areas (MPAs) or reserves operating co-operatively and synergistically, at various spatial scales and with a range of protection levels that are designed to meet objectives that a single reserve cannot achieve” [19]. This definition is clearly open to interpretation. How can we accurately assess whether an MPA network is fulfilling its specific objectives, and how does this compare to the methods used to assess whether a single MPA is effective?

It can be difficult to identify attainable management goals for MPA networks—and to design a process for evaluating whether they achieve those goals—without a clear understanding of their objectives or potential functions. Different types of networks exist based on varying management needs and goals. Theoretical studies address optimal size and spacing of protected areas to integrate population connectivity into the design of MPA networks (e.g., [17], [20], [21]), but there is no empirical evidence for the predicted outcomes of either existing networks or those under development. Recently, we proposed an analytical framework for assessing whether ecological effects across an entire network are greater than the sum of the ecological effects that occur within each MPA in the network [22]. In this paper we provide (1) a brief review of this analytical framework for network effects along with the definitions of different network types to clarify what can realistically be expected from each; and 2) to our knowledge, the first analysis that evaluates an MPA network effect, using monitoring data from a MPA network in Hawai'i.

## Monitoring Networks Based on Expected Outcomes

MPA networks can vary in their objectives, which should be explicitly considered to evaluate whether management goals are being met [23], [24]. However, complex scenarios are not conducive to the development of a single, one-size-fits-all assessment. Appropriate targets can vary based on situation-dependent criteria such as marine habitat distribution, the life-history traits of species targeted by management, and the socio-economic and cultural context in which the network is established. How can we assess the effectiveness of an MPA network at achieving a set of specific objectives? First, we must understand the different types of networks and the potential outcomes and limitations of each type.

We have defined five different types of “networks” that represent different goals and intended outcomes from collections of marine reserves and other MPAs (Table 1), [22]. Briefly, a network of MPAs could be an *ad-hoc* or *regional network*, a grouping of MPAs that are in proximity to each other but were not planned as a synergistic network; a *conservation network*, designed to have strict conservation goals in order to conserve the representative ecological characteristics of an area or ecosystem by protecting replicated sites that encompass habitats or species of interest; a *management network*, which manages and facilitates the economic uses of marine resources at a broader scale than a single-MPA approach would have afforded; a *social network* based on human interactions across groups of people including MPA managers, stakeholders, decision-makers, and scientists who transfer knowledge, share best practices, and build capacity; or a *connectivity network*, a set of multiple marine reserves and other MPAs designed *a priori* to be connected by the dispersal of larvae and/or movement of juveniles and adults, whose general goal is to maximize conservation and/or fisheries benefits from no-take areas. Examples of each of these network types can be found worldwide (Table 1). However, population connectivity is integral for effectively achieving the goals of protecting an adequate portion of a region (regional network), a particular species, group of taxa, or habitat (conservation network), and an assemblage of fished species that are harvested in areas outside the network to benefit local fisheries and the communities they support (management network). Thus, we assert that population connectivity should be a fundamental goal of network design and establishment to meet ecological goals.

**Table 1:** Definitions, goals, and examples for each type of marine protected area (MPA) network.

MPA network type	Definition	General network goals	Example network(s)
Ad-hoc or Regional	An unplanned collection of MPAs in a given area, not established with a cohesive goal	To meet international conservation targets, serve as potential foundation for a planned network	North-western Mediterreanean, Hawai'i, Caribbean
Conservation	A collection of MPAs in a given area aimed at protecting conservation priority sites	To protect replicates of representative ecosystems, critical areas, damaged habitats	Great Barrier Reef, Chile, Australian Commonwealth MPA networks, Florida Keys
Management	A collection of MPAs in a given area established to manage a marine resource and multiple human uses	To protect targeted species, increase reproductive capacity, increase yield, optimize coastal uses while meeting conservation targets, avoid conflicts	West Hawai'i, US West Coast Rockfish Conservation Areas, US Essential Fish Habitat Closures
Social	A collection of MPAs whose managers, practitioners, stakeholders, decision-makers, scientists, and others interact and transfer knowledge	To promote interaction among participants to effectively plan, manage, implement, or monitor area-based management of marine resources and associated uses	Mediterreanean Protected Areas Network (MedPAN), Caribbean Marine Protected Area Managers (CaMPAM)
Connectivity	A set of multiple MPAs connected by the movement and dispersal of larvae, juveniles, or adults	To maximize conservation benefits but minimize no-take area by establishing multiple, interconnected MPAs	Papua New Guinea, Gulf of California, California coast, Moorea, West Hawai'i

A properly designed connectivity network should ensure that it is not merely establishing a disconnected collection of single reserves and other types of MPAs, but that it instead protects a set of sites that allow connections among populations within protected habitats and ecosystems. Population connectivity includes not only larval dispersal, but also movement of juveniles and adults, which can augment the increased benefits provided by MPA networks as long as fishing mortality encountered while moving between MPAs does not negate the benefits provided by individual reserves [25], [26]. Key considerations for a connectivity network also include appropriate coverage across a geographical gradient and expected economic outcomes for managed fisheries in the surrounding waters (e.g., [27], [28], [29]). Below, we summarize an analytical framework for evaluating whether a connectivity network is effectively meeting the goal of increasing the density, production, or fishery yield of targeted species.

## An Analytical Framework for Monitoring Networks

The literature includes many papers on the proper design and evaluation of marine reserves and other types of MPAs (e.g., [28], [30]) as well as design criteria for incorporating connectivity into network design (e.g., [20], [31], [32], [33]) but there are very few papers that provide guidance on the evaluation of an MPA network (e.g., [34]). Those tasked with monitoring a network of MPAs must know how to measure whether that network is effective in meeting its goals. When focusing on connectivity networks, the key question is whether there is a significant overall network effect that is greater than the sum of the individual MPA effects. We test if the magnitude (or effect size) of a response (e.g., greater biomass of a target species inside MPAs relative to outside the MPAs) at the scale of the network (*ES*_network_, i.e., both inside and outside MPAs) is greater than the sum of the magnitudes of change occurring in individual MPAs (*ES*_MPA_). In other words, there is a synergistic interaction occurring among protected areas, such that:
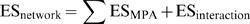
where *ES_interaction_* is the magnitude of the interaction effect for the MPAs within a network, and the hypothesis to be tested is:
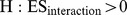



The evaluation of this effect would require both consideration of the overall goals of the network, which vary with the type of network [22], and data from a rigorously designed monitoring program at the appropriate scale with suitable controls at both local and regional levels. Based on this mathematical framework, we use data from the islands of Hawai'i and Maui to illustrate one of the first MPA network-wide analyses.

## Network Analysis: Hawai'i

We tested whether a planned network of MPAs, established in 1999 on the west coast of the island of Hawai'i (hereafter West Hawai'i), exhibited a management response consistent with a network effect. The nine MPAs in West Hawai'i, combined with eight relatively small preexisting protected areas, collectively protect 35% of the coast from fishing by the aquarium trade [35]. The Hawai'i aquarium trade most heavily exploits the yellow tang (*Zebrasoma flavescens*) [36], a common fish on Hawaiian reefs and an ecologically important herbivore [37]. Although yellow tang can live over 40 years, the prime target size range for aquarium-trade yellow tang corresponds to fish ≤2 years [38]. Reports of illegal fishing in the network are rare; MPAs are close to shore and poachers' activity is easily spotted [37]. Due to protection from fishing, increases in yellow tang abundance are evident in these MPAs [39]. In addition, there is large-scale population connectivity of yellow tang within the West Hawai'i MPA network and evidence of larval seeding to unprotected areas [40], demonstrating that these MPAs function as a connectivity network.

We compared yellow tang abundance in the planned West Hawai'i network to yellow tang abundance in two marine reserves on Maui (‘Ahihi-Kina'u, established in 1973, and Molokini, established in 1977). These two reserves were implemented based on separate management processes and were not planned to be ecologically connected. Although similar connectivity studies have not been performed on Maui, the lack of replicated MPAs across the dispersal distance of yellow tang illustrates the lack of network connectivity planning for the two separate Maui reserves. In contrast to the West Hawai'i MPAs, which only limit aquarium collecting and mostly protect herbivores, no-take reserves on Maui provide protection for all species. However, the small size of most of Hawai'i's protected areas limits their effectiveness for larger, more mobile predators [41]. Thus, there are likely few significant trophic cascades that can cause major differences between MPAs in West Hawai'i and marine reserves on Maui in terms of yellow tang abundance. Poaching is considered to be rare in Maui marine reserves [42].

**Figure 1:**
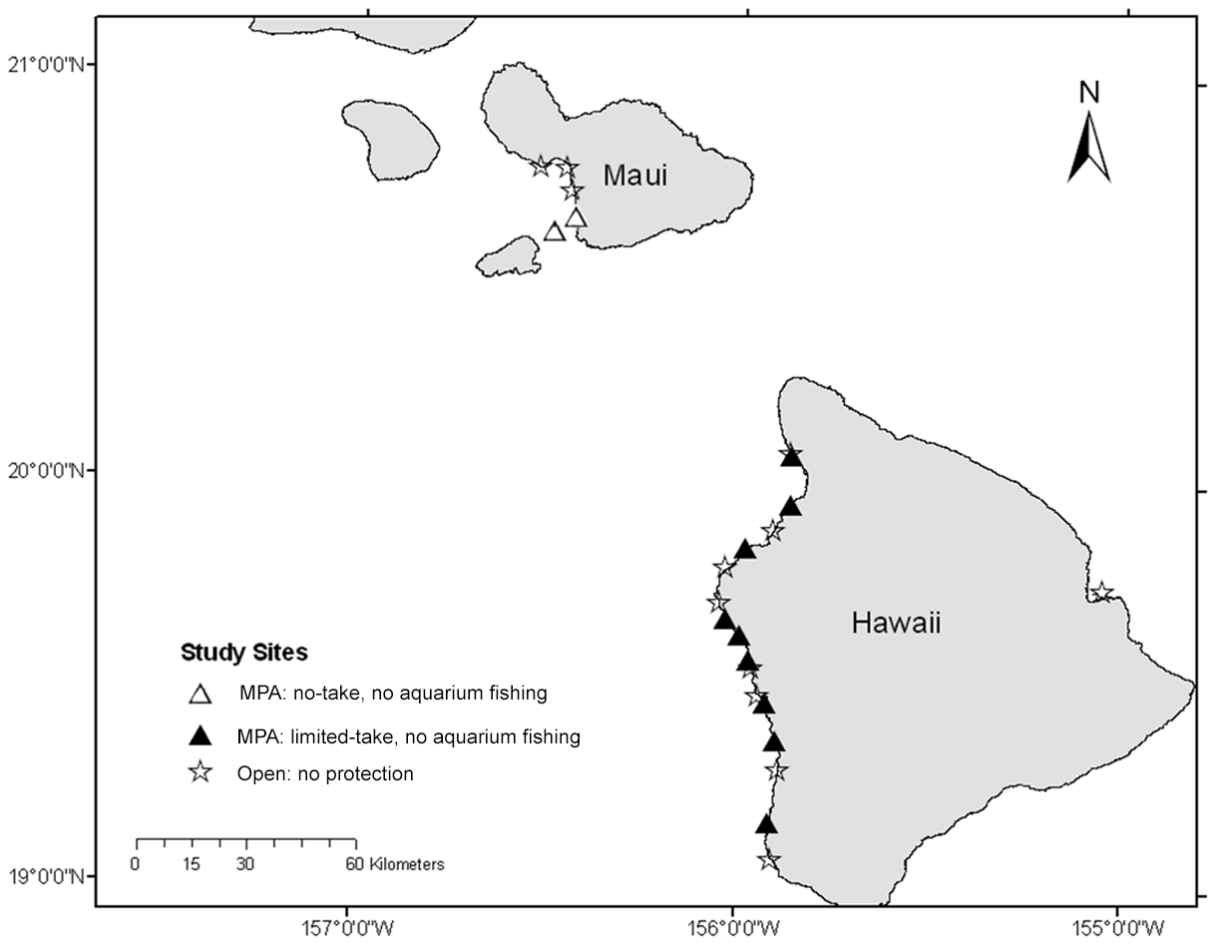
Map of study sites inside and outside protected areas in West Hawai'i and Maui.

Although Maui has one additional marine reserve (Honolua-Mokulèia Bay, located ∼65 km from the nearest marine reserve at Molokini), which contributes to the total protected area representing <1% of the coast, only ‘Ahihi-Kina'u and Molokini were monitored during time frames that overlapped with the West Hawai'i MPAs. On both islands, study sites were selected for the analysis based on similar monitoring methods during 2000–2003, except for Molokini, which included data from 1996–2004 [39]. After 2004, monitoring of the ‘Ahihi-Kina'u and Molokini reserves ended, while sampling of the West Hawai'i MPAs is ongoing. At each island “control” study sites open to all fishing activities (nine in West Hawai'i and three on Maui) were monitored during the same time period (Figure 1).

Sampling methods are detailed in Tissot et al. [39], but briefly, fish densities for both yellow tang and potential predators were estimated via visual search by a pair of divers along four 25×4 m strip transects per site. We estimated densities of top and mid-level predators using predator groups previously identified for Maui and West Hawai'i [43], excluding those that are known not to prey on Acanthurids such as yellow tang (i.e. the introduced snapper, *Lutjanus kasmira* or taape; [44], [45]. All sites were surveyed bimonthly, weather permitting, for a total of six surveys per site per year with the exception of logistical constraints in the summer of 2002. To be consistent among islands, we used 1999–2000 as a “before” MPA survey period (1996–2000 for Molokini) and 2001–2003 (2001–2004 for Molokini) as an “after” MPA implementation survey period.

To test for a network effect, we first conducted a three-way repeated-measure ANOVA with nested study sites after diagnostic tests confirmed the assumption of equal variances. The statistical model for the analysis is:
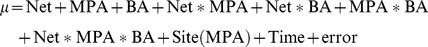



Where, **μ** denotes the population mean, **Net** the partitioned variability between network and non-network locations, **MPA** the partitioned variability between MPAs and open areas, **BA** the partitioned variability between before and after time periods, **Site** the partitioned variability among replicated study sites nested within MPAs, and **Time** the surveys as a random repeated measure. The random repeated measures accounted for the yearly averages of yellow tang density at each site and corrected for the non-independence in yearly sampling by site. We accounted for differences in the total area protected by the West Hawai'i sites and Maui sites by using ratios of before-after, outside-inside, and networked and non-networked yellow tang densities.

We predict that a significant network effect would be indicated by a greater increase (before versus after) in abundance in MPAs (and perhaps open areas) around an island with a network than on one without a network. This result would be indicated statistically by any significant first or second order interaction involving the factors **Net** and **BA**, and a significant effect size of the network. An effect size of a network could be estimated as:
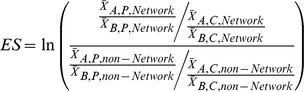
where 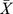 is the average density of yellow tang before (*B*) or after (*A*) the MPAs were established in the protected (*P*) or control (*C*) locations within the *Network* (e.g., West Hawai'i) or *non-Network* (e.g., Maui) conditions, which is tested for a statistical difference from zero using a z-test.

**Figure 2:**
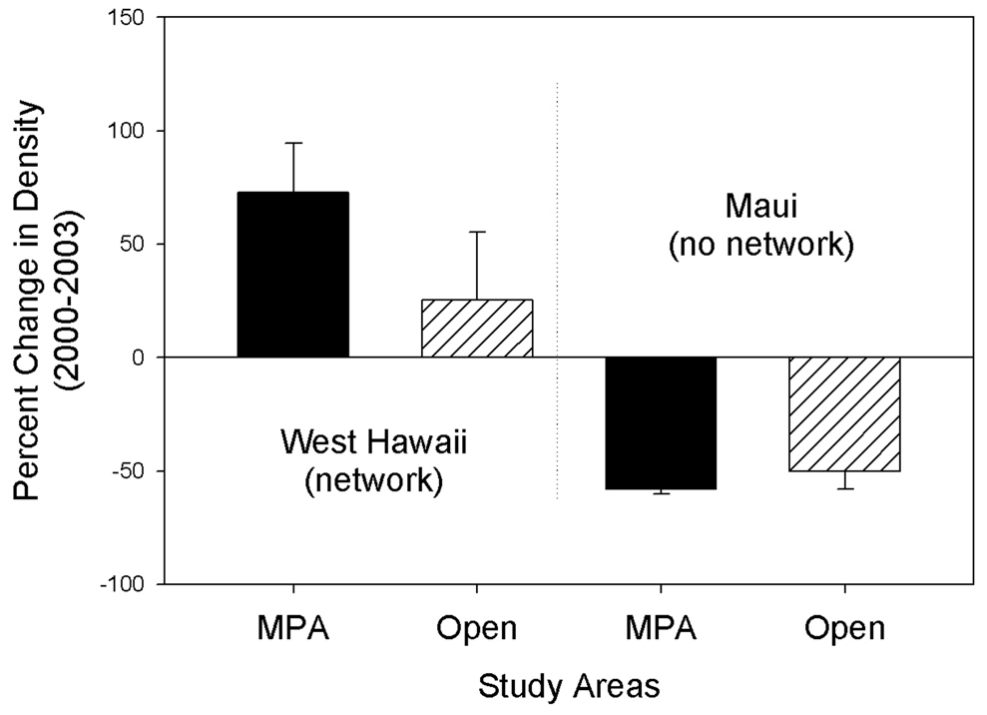
The mean percent change (±1 SE SE) in density of yellow tang (*Zebrasoma flavescens*) before versus after MPA network establishment in West Hawai'i within limited-take and no-take MPAs and control (open to fishing) sites on West Hawai'i and Maui.

The results of these analyses for the West Hawai'i and Maui data show significant Net*BA and Net*MPA interactions (Table 2). These results support the hypothesis that MPAs on West Hawai'i had significantly greater percent change in density within MPAs and open areas before vs. after network establishment compared to the Maui non-network sites during the same period (Figure 2). Moreover, the network effect size was significantly greater than zero (Mean network effectiveness  = 1.63±0.69 [95% C.I.], n = 18, z-test, p<0.001). Overall, yellow tangs within marine reserves and open areas on Maui declined during the study period, while both MPAs and open areas in West Hawai'i increased during the same time period. Although both marine reserves on Maui declined overall, abundances at ‘Ahihi-Kina'u during the study period were high but variable and yellow tang were relatively less common at Molokini.

**Table 2:** Three-way analysis of variance of yellow tang abundance on West Hawai'i versus Maui (factor Network), within and outside protected areas (MPA), before (1999–2000) vs. after (2001–2003) network establishment on West Hawai'i (BA), and interactions among all fixed factors (see text for details).

Source	DF	SS	MS	F	P
Network	1	1275	1275	6.90	0.009
MPA	1	4572	4572	14.01	0.001
BA	1	38	38	0.21	0.649
Network*MPA	1	908	908	4.91	0.028
Network*BA	1	1275	1275	6.90	0.009
MPA*BA	1	301	301	1.63	0.203
Network*MPA*BA	1	148	148	0.80	0.373
Site(MPA)	16	9212	57	3.11	<0.001
Time	5	1945	389	2.10	0.066
Error	235	43442	185		
Total	263	67390			

Replicated study sites for all treatments were nested within MPA and time was treated as a random, repeated measure factor.

Our data illustrate results consistent with a network effect that is greater than the sum of individual MPA effects. Although the West Hawai'i MPAs account for a greater area of protected coastline (35%) than the Maui marine reserves (<1%), a network effect is still indicated when the total area protected is standardized by response ratio (e.g. outside-inside comparisons). We compared protected sites that were both no-take for yellow tang, however the Maui sites were completely no-take marine reserves while some fishing for non-aquarium trade species is allowed in the West Hawai'i MPAs. To investigate whether differences in yellow tang predators may influence the densities of yellow tang across protection levels, we compared the top and mid-level predators by protection level (MPA, Open) and by island (Maui, West Hawai'i) (Table 3). For mid-level predators, the significant interaction term describing the effects of both island and protection level shows that there are in fact higher densities of these predators in open vs. MPA sites on Maui (Table 4). Overall there were no significant differences in densities of top predators when collectively comparing Maui and West Hawai'i. Thus, lower densities of yellow tang in Maui MPAs are not due to higher predator densities than the West Hawai'i MPAs. The length of protection for Maui marine reserves is greater than that of the West Hawai'i MPAs (established in 1973, 1977, vs. 2000, respectively). If the densities of yellow tang were greater in Maui reserves due to the longer timeline to achieve a response, we would expect our network effect size to be lower as a result. Yet we did not see a higher yellow tang response in the older Maui reserves but instead a higher response in the Hawai'i network. In addition, more recent data from West Hawai'i demonstrate that in 2012 yellow tang responses to MPA network protection were still as high or higher than those observed during the first five years after reserve establishment [46], indicating that the network effect we observed may in fact be a conservative estimate. Collectively, these data show that the West Hawai'i MPAs have functioned as a network based on the established management goals [39].

**Table 3:** The mean density (±1 SE) of top and mid-level predators on Maui and West Hawai'i under different management regimes.

	Mean density (100 m^−2^)	
	Open sites	MPA sites
**Top predators**		
West Hawai'i	0.01 (0.01)	0.04 (0.03)
Maui	0.39 (0.10)	0.08 (0.05)
**Mid-level predators**		
West Hawai'i	0.36 (0.02)	0.41 (0.02)
Maui	0.68 (0.11)	0.39(0.10)

Open  =  open to fishing. MPA =  marine protected area. Top predators: Carangidae, Carcharhinidae, Sphyraenidae; Mid-level predators: Aulostomidae, Lutjanidae, Muraeidae, Scorpaenidae, Serranidae, Synodontidae.

**Table 4:** Two-way analysis of variance of top predator and mid-level predator densities on West Hawai'i and Maui (factor Island), within and outside protected areas (MPA), and for the interaction among these fixed factors (see text for details).

Predator level	Source	DF	SS	MS	F	P
Top	Island	1	1.59	1.59	0.62	0.43
	MPA	1	0.78	0.78	0.31	0.58
	Interaction	1	1.12	1.12	0.44	0.51
	Error	117	299.6	2.56		
	Total	120	304.8			
Mid-level	Island	1	0.56	0.56	3.59	0.06
	MPA	1	0.35	0.35	2.24	0.14
	Interaction	1	0.72	0.72	4.57	0.04
	Error	117	18.4	0.16		
	Total	120	20.7			

Top predators: Carangidae, Carcharhinidae, Sphyraenidae; Mid-level predators: Aulostomidae, Lutjanidae, Muraeidae, Scorpaenidae, Serranidae, Synodontidae.

The ideal analysis for the network effect would involve similar types of comparisons to those made here but among MPAs that were created over the same time period and followed for longer lengths of time. These data gaps illustrate the importance of prioritizing and standardizing monitoring efforts given the financial and logistical resources at hand [47]. To that end, field monitoring coupled with simulation modeling is a powerful way to both generate data-driven hypotheses and target the monitoring data most useful for detecting a network effect [48].

## The Value of Interactive Empirical-Modeling Approaches

From a practical point of view, a monitoring program that provides the full complement of data to evaluate an MPA network effect can be difficult and cost-prohibitive. Quantitative monitoring of algae, fishes, and invertebrate densities, fishing mortality, socio-economic indicators, and measures of larval retention within and connectivity among MPAs [49] should be evaluated both before and after MPA establishment, from areas inside MPAs and in unprotected areas *inside* and *outside* the network—all while incorporating sufficient sample replication, avoidance of spatial confounding, and appropriate temporal replication [22], [50]. Given the time and energy required to monitor a single MPA, let alone an MPA network, the logistical and financial considerations associated with such an endeavor would be high. Further, the reality of having access to a full network that is designed to function as a connectivity network, coupled with access to another comparable control “non-network” not designed for connectivity, is unlikely.

To prioritize data collection given limited resources, and to further refine the empirical approach discussed above, predictions generated from spatially explicit models can serve as model experiments to generate hypotheses and test the effectiveness of an MPA network [16], [20], [48]. One can ask how the ecosystem and fisheries responses would change if one or more protected areas were poorly connected to the rest of the network and then compare how both the individual MPAs and the network as a whole respond to different levels of connectivity. These predictions can be vital for estimating the timing and magnitude of expected MPA effects by quantifying the separate contributions of reduced fishing mortality and potential network effects, identifying potential spillover effects in references areas, characterizing the relative importance of multiple factors affecting network success, and ultimately refining future monitoring plans to inform adaptive management of the network. For example, during the process to design an MPA network mandated by California's Marine Life Protection Act (MLPA), three-dimensional ocean circulation models were coupled with spatially explicit models of the dynamics of fished species and fishing effort to evaluate the projected impact on abundance and fishery yield of all submitted MPA designs [6], [51], [52]. These spatially explicit metapopulation models use estimated larval dispersal kernels, habitat distribution, and fishing mortality (inside and outside reserves) to predict spatial and temporal patterns of larval production and replenishment, which in turn predict resulting geographic patterns of adult abundance, size structure, biomass and spawning potential, and levels of population sustainability [6], [53]. Empirical estimates of abundance, size structure, and recruitment rates derived from monitoring studies, such as those illustrated in our Hawai'i example above, are critical for both parameterizing these population models and evaluating the model predictions.

The design of monitoring programs can also benefit from these model predictions. For example, models can predict not only the rate and magnitude of change in response variables but also the likely degree of associated spatial and temporal variability [46], [47], [54], [55]. Given this information, monitoring can optimally allocate sampling effort to sampling designs that increase the likelihood of detecting responses empirically (i.e., by identifying the timing, number, and distribution of spatial and temporal replicates). This iterative approach provides a powerful tool for adaptive management when empirical data from monitoring informs models and, in turn, model results further refine monitoring designs [46].

## Conclusions

As more MPA networks are established, it becomes increasingly important to implement thoughtful and rigorous monitoring plans to assess their effectiveness [24]. The benefits of connectivity underscore many network management goals, yet the other types of networks defined here can accomplish their own set of protective objectives (Table 1). Thus, it is essential to carefully consider which network type(s) is most in line with the stated goals. Thorough monitoring designs should be outlined prior to network establishment, accounting for outside, inside, control, and experimental MPA sampling across an appropriate spatial and temporal scale and prioritizing the data types most critical for assessing network effects. Our example of MPAs in West Hawai'i and Maui illustrates the need for well-balanced data in order to effectively determine whether a network effect is occurring. In reality, there are many logistical constraints on MPA monitoring, which highlight the value of coupling models with empirical data to generate network-level hypotheses and evaluate the responses to protection in a network. We encourage regional management bodies, scientists, and stakeholders to discuss all of these key components as a regular part of the network planning process and to work collectively to develop optimal monitoring strategies given the available resources.
